# Clinical efficacy of therapeutic keratoplasty using corneal xenografts in patients with corneal ulcers

**Published:** 2019

**Authors:** Mykola Turchyn, Mariya Marushchak, Inna Krynytska, Ivan Klishch

**Affiliations:** * Otolaryngology and Ophthalmology Department, I. Horbachevsky Ternopil State Medical University, Ternopil, Ukraine; ** Functional and Laboratory Diagnosis Department, I. Horbachevsky Ternopil State Medical University, Ternopil, Ukraine

**Keywords:** corneal ulcers, keratoplasty, corneal xenograft

## Abstract

**Objective.** to assess the clinical efficacy of keratoplasty using a corneal xenograft in patients with corneal ulcers of various etiologies.

**Methods.** A total of 46 patients (49 eyes) with complicated corneal ulcers (perforated or with impending perforation) have been operated. Freeze-dried corneal xenografts have been used for keratoplasty.

**Results.** Our results showed that postoperative period after xenogeneic keratoplasty in patients with corneal ulcers was uneventful and the transplant underwent gradual resorption. In all patients with non-infected corneal erosions, ulcers healed completely and corneal fistulas were fully closed. The implanted corneal xenograft undergoes complete resorption between 2 to 3 months.

**Conclusions.** Given the shortages of donor material, the demonstrated efficacy of using corneal xenografts in patients with complicated corneal ulcers requiring therapeutic keratoplasty allows recommending corneal xenografts for wide use to relieve inflammation and to preserve the eye.

## Introduction

Corneal disorders are one of the leading causes of blindness and impaired vision. According to WHO, they are among the three principal factors of loss of sight, accounting for 6.6% to 39.3% of the cases [**1**]. Corneal damage results from injuries and burns, the latter known to be the most severe of all vision-related problems. In part, corneal injuries account for 29% of the primary disability in people of productive age, and the incidence of eye burns is from 6.1 to 38.4% of all eye injuries, according to various authors [**2**,**3**]. 

Regardless of recent advances in ophthalmology, including medical and surgical treatments, 40% of the injured become permanently disabled; a significant proportion of them are young people of productive age. Septic corneal ulcers are a problem of no smaller significance, accounting for 36.6% of external ocular problems [**4**]. To a large degree, this is because traditional medical treatments are incapable of reducing the complications of eye injuries beyond certain limits. Generally accepted strategies of medical management in eye burns and corneal ulcers are often far from being effective [**5**,**6**]. The insufficiently high efficacy of medical treatments is often related to impaired reparation and regeneration, which often leads to perforation and eye loss. This is why corneal conditions often call for urgent surgical interventions, especially in progressive lysis and impending corneal perforation. The above suggests that adequate management of patients with eye injuries and corneal ulcers remains a current medical and socio-economical issue.

One of the promising ways to manage patients with eye injury and corneal ulcers includes the technology of keratoplasty. Given the high regenerative capacity of corneal tissue, most clinicians tend to acknowledge the benefits of surgical treatment in this patient population, with biological coating being a backbone therapeutic modality. Various donor-derived materials are known to be used for this purpose, including cornea, sclera, dura mater, fasciae, amniotic sac, etc. Although corneal transplantation has been known for approximately one hundred years and currently is the most frequently used therapeutic technology, the difficulties with its implementation have constituted the essence of the scientific and applied challenges of modern ophthalmology to this day. These challenges include the difficult procurement of sound, high-quality donor material, the imperfect technique of surgical keratoplasty, immunological incompatibility between the graft and the host tissues, shortcomings of legal groundwork, etc. [**7**]. The above-mentioned issues have become an impetus for exploration of promising technologies for manufacturing and using xenogeneic donor material.

The profound understanding of biology of corneal tissue juxtaposed with known progress in microsurgical technology (especially taking into account the positive experience with anti-inflammatory drugs) currently serve as a rationale behind the success of keratoplasty, the latter being a recognized current modality to counter the blindness caused by corneal opacification [**8**]. 

A promising approach to above challenges included a technological method developed and implemented in Ukraine under the guidance of Prof. V.V. Biguniak, which involved the use of cryo-lyophilized xenogeneic biomaterial, specifically, porcine skin (a material widely used to treat patients with burns) [**9**]. In view of the positive outcomes of using xenogeneic graft material, the manufacture and ophthalmologic uses of corneal grafts based on porcine corneal tissue are currently viewed as obviously promising. At that, there remains a challenge of providing modern ophthalmology with an optimal preserved biograft, that is, a corneal xenograft.

The aim of the work was to assess the clinical efficacy of keratoplasty using a corneal xenograft in patients with corneal ulcers of various etiologies.

## Methods 

*The manufacturing technology of corneal xenografts*. The technology to obtain donor material included harvesting the corneas directly on the kill floor [**10**], in compliance with the rules of the European Commission for supervision of laboratory and other experiments with animals of various species used for scientific purposes. The separated corneas were rinsed in sterile distilled water and after that, they were immersed into process solutions. Prior to cryopreservation, the corneas were processed with a cryoprotectant of the following composition: lactose 11.5 mL, glycerol 5 mL, egg yolk 20.0 mL and 100 mL of distilled water (warmed to 40°C). In the protective solution, the corneas were straightened; then, air was removed from the bags and the bags were pressure-sealed. To equilibrate the material, the bags were placed into a refrigerator for 3 hours at a temperature from +4 to +6°C (inclusive). At the next step, the bags were placed into vapors of liquid nitrogen for 10 minutes at –120°C with subsequent transfer to a tank with liquid nitrogen [**11**]. Prior to lyophilisation, freeze dryers and spreading racks were treated with 96° ethyl alcohol and trays/retainer nets were sterilized in an autoclave. Then xenogeneic corneas were spread out on a tray and fixed with a stainless steel mesh. After that, the free rack was immersed into the freeze dryer, the refrigeration unit was turned on, and the temperature was brought to –30 –35°C (inclusive). Then the trays were loaded with the racks where xenogeneic corneas were placed. The temperature conditions in the drying chamber were monitored using a temperature detector. Vacuum pump was activated to reduce the temperature to –45 C in a 4 Pa vacuum. The unit ran for 3 hours in the above mode; after that, a rack heating system was activated and the temperature was brought to +20°C. After 4 hours of operation, the temperature in the freeze dryer was brought to +45°C. The entire lyophilisation process was performed under instrument monitoring; the findings of the monitoring were documented in the tissue drying protocol. 

**Fig. 1:**
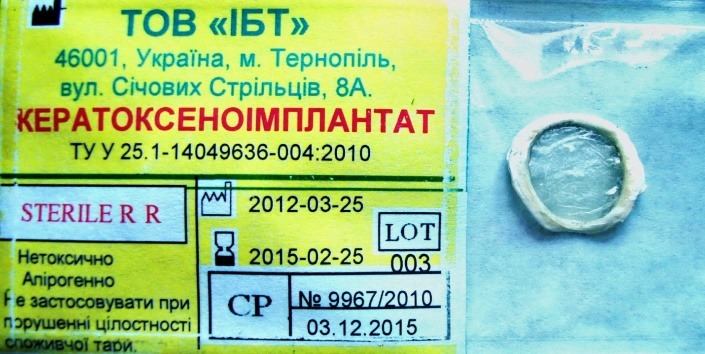
Lyophilized corneas available as freeze-dried corneal xenografts Notes. Translation of the label: IBT LLC, 8A Sichovykh Striltsiv Str., Ternopil, 46001, Ukraine. CORNEAL XENOGRAFT TU U Standard 25.1-14049636-004:2010 Sterile R R. Non-toxic. Pyrogen-free. Do not use if consumer packaging has been compromised. 2012-03-25. 2015-02-15. LOT 003. CP No.9967/2010|03.12.2015

The lyophilized corneas preserved in accordance with the above technique as freeze-dried corneal xenografts were then placed in polyethylene bags and packaged (**Fig. 1**). 

**Fig. 2:**
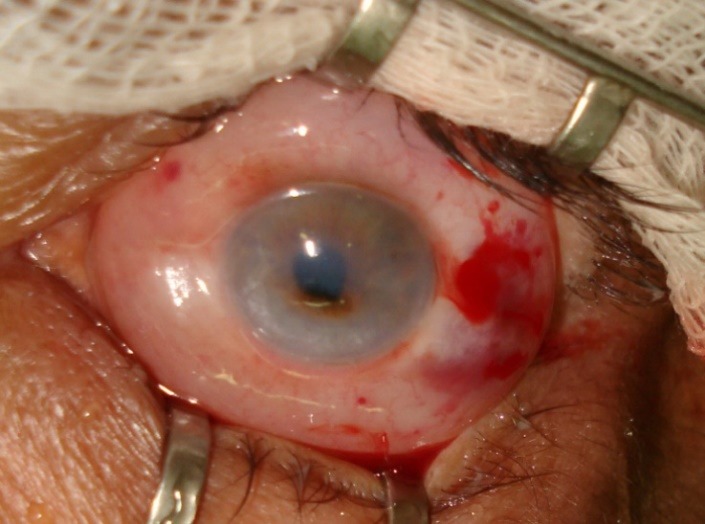
Perforation in the center of a patient's cornea (a complication of corneal ulcer). Preoperative status

The clinical study was conducted in the Ophthalmology Department of Ternopil University Hospital (Ternopil, Ukraine) on patients treated between 2010 and 2014. A total of 46 patients (49 eyes) with complicated corneal ulcers (perforated or with impending perforation) have been operated (**Fig. 2**).

Surgery was considered to have a positive outcome in the following circumstances: ulcer healing was attained, corneal perforation was prevented, and, in case of perforation, the eye(s) and light perception were preserved (if present preoperatively).

The results were processed with methods of mathematical statistics using Excel software.

## Results

**Fig. 3:**
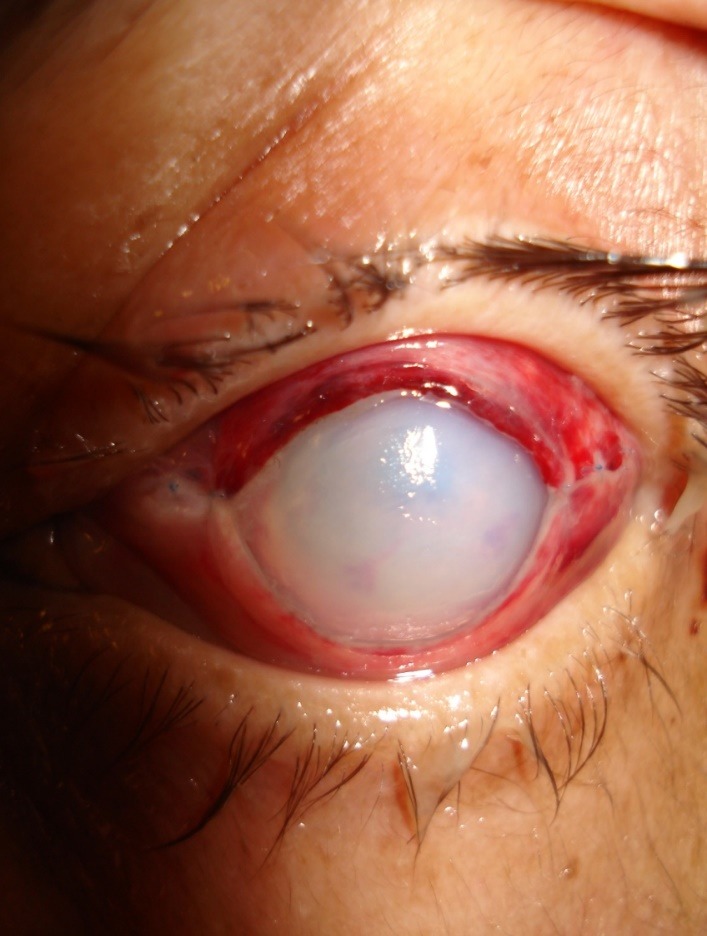
Corneal status in early postoperative period after therapeutic xenogeneic keratoplasty (heterokeratoplasty), Day 2 postoperatively)

In early post-operative period, all patients had a moderate inflammatory response and the edges of their post-operative wounds have adapted completely (**Fig. 3**). Resorption of corneal xenograft lasted from 3 weeks to 8 weeks. Already during the first 3 to 4 post-operative days, the operated eye was no longer painful on palpation, intraocular pressure returned to normal, and the anterior chamber (which was absent preoperatively) was restored.

**Fig. 4:**
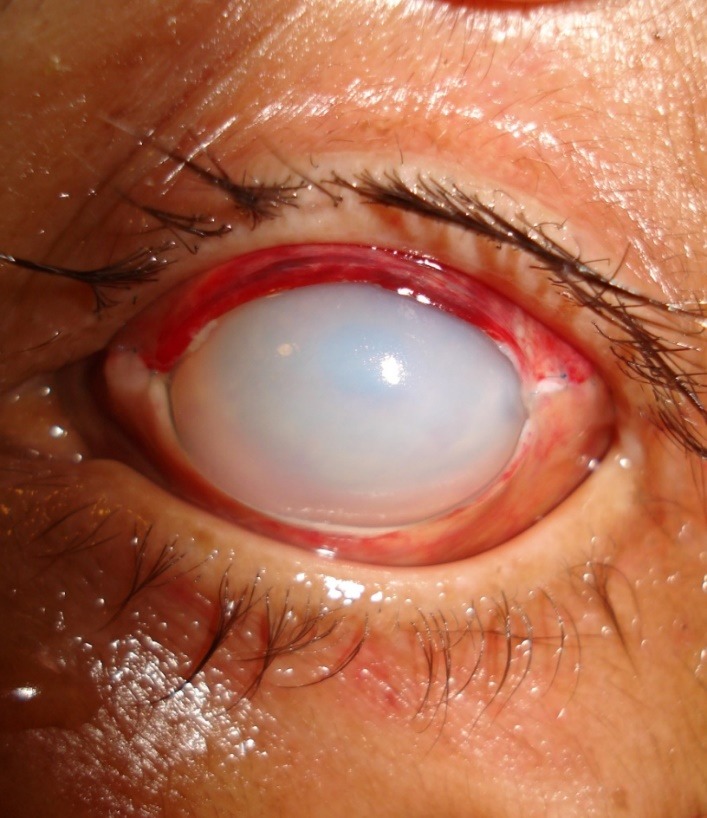
Corneal status in early postoperative period after therapeutic xenogeneic keratoplasty (heterokeratoplasty), Day 5 postoperatively)

At the time of discharge (Day 5), surgical outcomes in all 60 patients (69 eyes) included closure of the fistula with a corneal xenograft and a significant reduction in ocular inflammatory response (**Fig. 4**). 

**Fig. 5:**
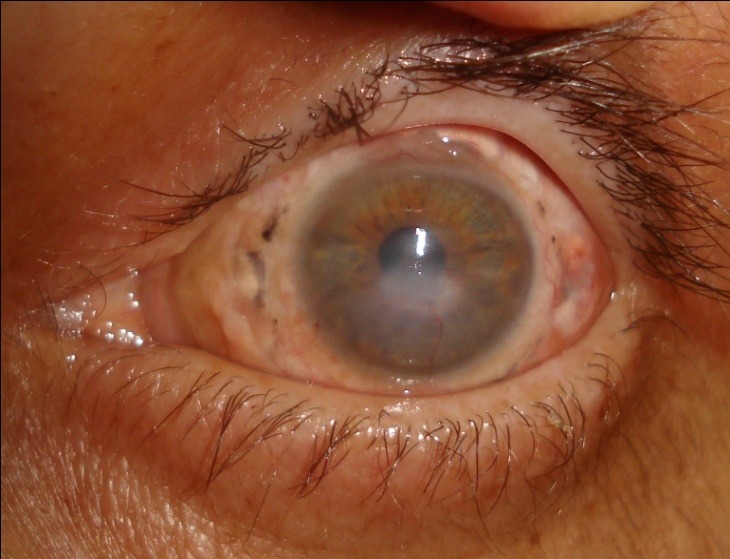
Corneal status 2 months after therapeutic heterokeratoplasty

The findings of patient follow-up (1.5 to 2.0 months postoperatively) demonstrated complete healing of ulcers and closure of corneal fistulas, with corneal opacifications at the sites of defects (with moderate vascularization). Sparing cornea from perforation and preservation of eyeball integrity and light perception (if present preoperatively) was possible in all patients with complicated corneal ulcers; this created further prospects of restoring patient's sight (**Fig. 5**).

In isolated instances, we observed conditions that required the repeating of keratoplasty. Thus, in 2 months, one eye (2.0%) had a lysis of corneal xenograft, which required the repeating of keratoplasty using a corneal xenograft; two eyes (4.1%) required the repeating of keratoplasty with a corneal xenograft in 1.5 and 10 months, respectively, which was also caused by a partial lysis of the graft. 

## Discussion

According to WHO, blindness secondary to corneal disorders is one of the leading causes of loss of sight [**12**]. Each year, 1.5–2.0 million cases of monocular blindness secondary to various corneal lesions are documented worldwide [**13**,**14**]. The most frequent causes of corneal lesions include keratitis and corneal ulcers [**15**]. Concerning corneal ulcers in particular, the available clinical experience showed that they are a group of rare but very serious conditions, which are difficult to manage. For example, according to a number of authors, anatomical destruction of the eye is the ultimate outcome in 8-9% of the cases, and in 17% of the cases, enucleation is undertaken due to lack of treatment effect [**13**]. Corneal damage leading to corneal ulceration occurs as a combination of direct microbial invasion and a release of collagenases, secondary to host chemotaxis of leukocytes [**16**].

Medical management of corneal ulcers does not always produce a therapeutic effect; this is due to a number of causes, such as the virulence of the pathogen, the secondary changes in corneal tissue (caused by polymorphonuclear infiltration of the cornea, activation of proteolytic enzymes and impaired reparation/ regeneration, with all these factors aggravated by destruction of tissues). These factors ultimately cause corneal perforation and eye loss [**8**,**17**]. This is why such corneal conditions call for emergency surgical interventions, especially in a setting of progressive lysis of corneal tissue and impending perforation. The available surgical techniques include conjunctival autografts [**18**], cyanoacrylate tissue adhesive [**19**], corneal–scleral–conjunctival transpositions and lamellar keratoplasty [**20**] and penetrating keratoplasty [**21**]. The use of tectonic corneal grafts has also been discussed [**22**]. Such approaches often place high demands on surgical skills and equipment; procurement and storage of donor tissues can also be an issue.

Clinical corneal transplantation is quite successful, especially in complication-free patients with non-vascularized native corneas [**23**]. At the same time, common complications of keratoplasty include graft rejection, corneal graft melting, secondary glaucoma, recurrence of primary disease, etc. [**24**]. Ideal biografts for corneal defect repair should meet strict specifications in terms of optical clarity, support of epithelial migration and adhesion, permeability to solutes, and stability to corneal proteases [**25**].

To date, the most widely accepted method of ophthalmological treatment is transplantation of native donor cornea. There is a continuously increasing demand for transplantation material, which is associated with an acute shortage of donor tissues in many countries due to demographic problems, increased incidence of infectious diseases, and a wider use of refractive laser surgery. Moreover, imperfect legislation and religious factors also contribute to this deficiency [**26**]. The above problems are driving the search for new ways to provide material for reconstructive corneal procedures.

Recently, there has been a resurgence of interest in potential use of corneas from non-human species, especially pigs, for human transplantation (xenografting) [**27**]. Xenotransplantation with fresh porcine corneas is viewed as a feasible alternative to the scarcely available human donor corneas [**28**].

There have been several attempts to use animal corneas for human transplantation, the first ones in the early 19th century [**29**]. These attempts involved corneas from pigs, sheep, dogs and rabbits [**30**], and, in the 20th century, corneas from gibbons, cows, and fish [**31**-**33**]. Although technical success was reported in several xenotransplantations, most of the xenografts from the dissimilar species were rejected during one month's time.

There are multiple anatomical and physiological similarities between pigs and humans as species [**23**,**34**]. Pigs are widely bred for the food industry, and they are available in large quantities. Additionally, they are much more cost-effective and less likely to transmit new pathogens to humans when compared to primates [**35**]. From a bioethical standpoint, porcine material is an acceptable alternative for transplantation in humans [**36**].

In 2014, Lee et al. aimed to investigate the opinion of Koreans awaiting a cornea transplant on the use of porcine corneas as a cure from visual loss. Demographic factors were shown to influence their opinion, with both elderly and well-educated people expressing less concern about the procedure than other people. Overall, 42.4% of the participants expressed favorable views regarding xenocornea transplantation [**37**]. 

Immunologically, however, nonhuman primates may appear preferable over pigs due to a larger genetic distance between pigs and humans. However, since the cornea is an immune-privileged tissue and lacks immediate blood supply, its use as a xenogeneic transplant is likely to be much more successful than that of organ transplants [**38**]. However, the recognition of galactose α1-3 galactose trisaccharides present on the surface of wild-type pig cells by human antibodies inevitably results in hyperacute rejection of the graft [**39**]. Additionally, safety concern regarding porcine endogenous retroviruses (PERVs) has been raised upon discovery that PERV can infect human cells in vitro [**40**].

There are numerous literature reports on experimental corneal xenografting in models using large- and small-sized animals. However, there have been but few recent studies of corneal transplantations from pigs to nonhuman primates with potential human relevance. Pan et al. [**41**] conducted a study of wild type (i.e., unmodified) porcine corneal transplants to rhesus monkeys. Untreated (non-immunosuppressed) monkeys rejected the grafts within 15 days, although no hyperacute rejection was seen. The rejected xenografts had inflammatory cellular infiltrates in the anterior stroma; the endothelium was either absent or abnormal. This suggested that corneal endothelium could be a principal target for antibodies or complement from the aqueous humor or for immune cells including T-cells and macrophages.

Our results showed that postoperative period after xenogeneic keratoplasty in patients with corneal ulcers was uneventful and the transplant underwent gradual resorption. In all patients with non-infected corneal erosions, ulcers healed completely and corneal fistulas were fully closed. We attribute the significant anti-inflammatory effect of corneal xenografts to their positive adsorptive, antitoxic, plastic, metabolic and redox properties. Surgical keratoplasty using a corneal xenograft manufactured from freeze-dried porcine corneas provides a reliable repair of corneal defects reduces or eliminates inflammation with subsequent epithelization and restores the anatomical integrity of the eyeball. The implanted corneal xenograft undergoes complete resorption during 2 to 3 months.

On the other hand, the use of conjunctival keratoplasty (Kuhnt's technique - displacing the conjunctiva of the affected eye onto the corneal surface), attained complete ulcer healing in only 20% of the patients; the rest of these patients required the repeating of the surgical intervention. 

## Conclusion 

Given the shortages of donor material, the demonstrated efficacy of using corneal xenografts in patients with complicated corneal ulcers requiring therapeutic keratoplasty allows the recommendation of corneal xenografts for wide use to relieve inflammation and to preserve the eye.
